# Nodakenin Ameliorates Ovariectomy-Induced Bone Loss by Regulating Gut Microbiota

**DOI:** 10.3390/molecules29061240

**Published:** 2024-03-11

**Authors:** Chunxiao Liu, Jingyue Chen, Zijiao Wang, Yueyao Li, Yuanyuan Zhang, Guangyu Li

**Affiliations:** 1 College of Animal Science and Technology, Qingdao Agricultural University, Qingdao 266000, China; liuchunxiao01@caas.cn (C.L.); wzjdgg1022@163.com (Z.W.); yueyaolee2001@163.com (Y.L.); yuanyuanzhang28@163.com (Y.Z.); 2 Institute of Special Animal and Plant Sciences, Chinese Academy of Agricultural Sciences, Changchun 130117, China; 82101211228@caas.cn

**Keywords:** osteoporosis, nodakenin, gut microbiota, intestinal barrier, metabolomics

## Abstract

Disordered gut microbiota (GM) structure and function may contribute to osteoporosis (OP). Nodakenin has been shown to ameliorate osteoporosis; however, its anti-osteoporotic mechanism is unknown. This study aimed to further reveal the mechanism of the anti-osteoporotic action of nodakenin from the perspective of the microbiome and metabolome. An osteoporosis model was induced in mice through ovariectomy (OVX), with bone mass and microstructure assessed using μCT. Subsequently, ELISA and histologic examination were used to detect biochemical indicators of bone conversion and intestinal morphology. Using metabolomics and 16S rRNA sequencing, it was possible to determine the composition and abundance of the gut microbiota in feces. The results revealed that nodakenin treatment improved the bone microstructure and serum levels of bone turnover markers, and increased the intestinal mucosal integrity. 16S rRNA sequencing analysis revealed that nodakenin treatment decreased the relative abundance of Firmicutes and Patescibacteria, as well as the F/B ratio, and elevated the relative abundance of Bacteroidetes in OVX mice. In addition, nodakenin enhanced the relative abundance of *Muribaculaceae* and *Allobaculum*, among others, at the genus level. Moreover, metabolomics analysis revealed that nodakenin treatment significantly altered the changes in 113 metabolites, including calcitriol. A correlation analysis revealed substantial associations between various gut microbiota taxa and both the osteoporosis phenotype and metabolites. In summary, nodakenin treatment alleviated OVX-induced osteoporosis by modulating the gut microbiota and intestinal barrier.

## 1. Introduction

Osteoporosis (OP) is a condition characterized by systemic disruptions in bone metabolism, leading to decreased bone mineral density (BMD) and structural deterioration [1]. The main population that suffers from osteoporosis is postmenopausal women, where metabolic disturbances in bone homeostasis are triggered by aging or estrogen deficiency [2]. Due to the growing prevalence of aging worldwide, osteoporosis has emerged as a global public health issue. Currently, the prevention and treatment of OP mainly depend on lifestyle modifications and various types of pharmacologic interventions [3,4]. Nevertheless, osteoporosis treatment is sometimes hindered by the financial burden and adverse reactions associated with drugs [5], so there is an urgent need to develop more efficient and safer treatment options.

Gut microbiota can interact with various organs and systems of the body and are essential influencers of metabolism-related diseases [6]. Almost 10 trillion bacteria in the human gastrointestinal system can interact with the host and play a crucial role in influencing metabolic illnesses like obesity, diabetes, and osteoporosis [7,8,9]. Clinical studies have shown that gut microbiota can influence bone quality and strength [10,11,12]. In addition, recent studies have shown that estrogen deficiency can induce imbalances in the gut microbiota (GM), impair intestinal mucosal barrier function, and increase reactivity in the inflammatory system [13,14,15]. However, their complex mechanisms have not been completely clarified. Hence, the mechanism underlying the gut/bone axis deserves further investigation, and it would be interesting to search for an effective drug candidate that exerts anti-osteoporotic effects by modulating the gut microbiota.

Nodakenin, a furanocoumarin glycoside isolated from the roots of *Angelica sinensis*, has anti-inflammatory, antimicrobial, antioxidant, and antitumor effects [16,17,18,19,20]. It has recently been found that nodakenin can increase the subchondral bone volume and thereby reduce osteoarthritis [21]. We also demonstrated in our previous study that nodakenin alleviated osteoporosis by regulating bone formation and bone resorption [22]. Nodakenin exhibits significantly low oral bioavailability yet is efficacious. This indicates that nodakenin may exercise its pharmacological effects by modulating the gut microbiota and the resulting metabolites [23,24]. However, whether the mechanism by which nodakenin inhibits bone loss is related to changes in the gut microbiota has not been reported to date.

In this study, we used an OVX mouse model to mimic postmenopausal estrogen deficiency-induced osteoporosis. Furthermore, we utilized fecal microbiome and metabolome analysis to investigate how nodakenin alters the gut microbiota and its metabolite profile. We found that nodakenin treatment may ameliorate OVX-induced osteoporosis in mice by modulating the gut microbiota and repairing the gut barrier.

## 2. Results

### 2.1. Nodakenin Alleviates Osteoporosis in OVX Mice

**Figure 1:**
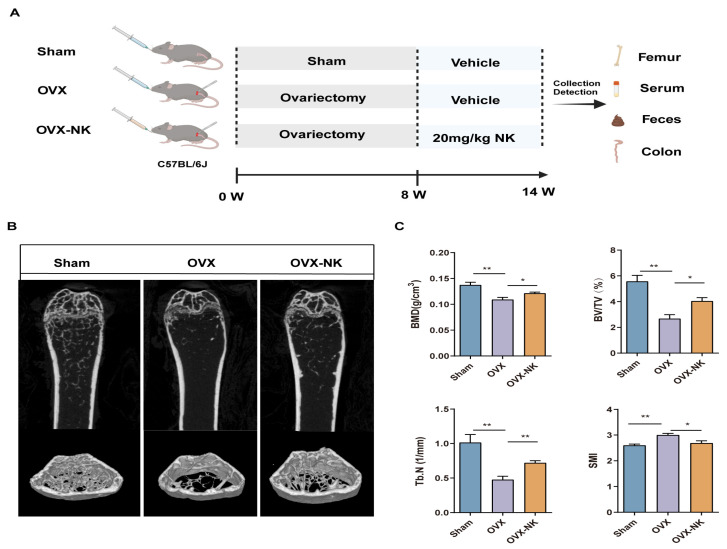
Effect of nodakenin on femoral bone microarchitecture in OVX mice. (**A**) Schematic diagram of in vivo experiments. (**B**) Micro-CT scanning representative image. (**C**) Statistics of bone morphometric parameters, bone mineral density (BMD), bone volume fraction (BV/TV), number of bone trabeculae (Tb.N), and structural modeling index (SMI). Mean ± SEM (*n* = 6). * *p* < 0.05; ** *p* < 0.01.

As demonstrated, we administered nodakenin medication for a duration of 6 weeks following an 8-week period of ovariectomy in mice. This was carried out to assess the impact of nodakenin on the bone loss generated by ovariectomy in mice with OP (Figure 1A). OVX mice gained significant body weight compared with the Sham group, while nodakenin treatment reduced the OVX-induced weight increase. There were no significant differences in the weights of the liver, kidney, and spleen (Supplementary Figure S1). μCT confirmed a significant decrease in BMD, BV/TV, and Tb.N and a significant increase in SMI in OVX mice compared to the Sham group, suggesting the successful modeling of osteoporosis in mice. BMD and bone microstructural parameters were significantly improved in the mice after nodakenin treatment (Figure 1B,C). It is suggested that nodakenin can alleviate OVX-induced bone loss.

### 2.2. Nodakenin Regulates Estrogen Deficiency-Induced Bone Turnover

**Figure 2:**
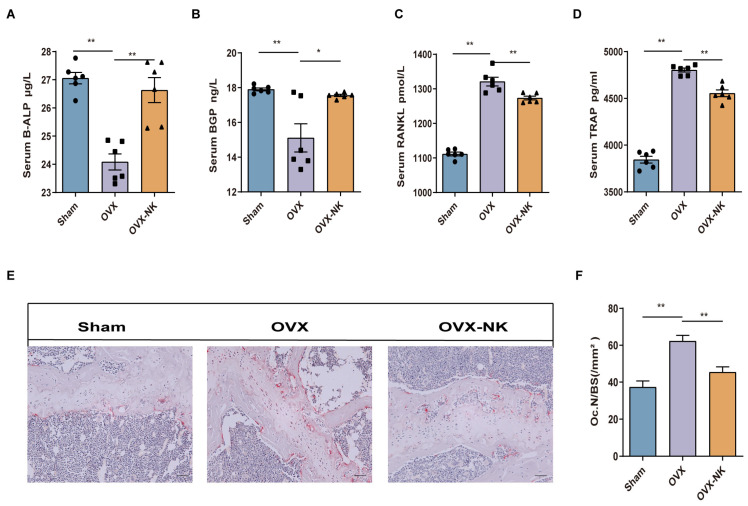
Nodakenin improves bone turnover marker expression in OVX mice. (**A**–**D**) Observation of the changes in B-ALP, BGP, TRAP, and RANKL in the serum of each group. (**E**) Representative pictures of TRAP staining. Scale bars are 100 μm. (**F**) ImageJ statistics of osteoclast number (Oc.N)/bone surface area (BS). * *p* < 0.05; ** *p* < 0.01.

In order to evaluate the impact of nodakenin on osteoporosis, we examined the changes in serum bone turnover marker levels via ELISA. The serum levels of the B-ALP and BGP bone formation markers were significantly elevated in nodakenin-treated mice compared to OVX mice, while TRAP and RANKL bone resorption markers were decreased (Figure 2A–D). In addition, we used TRAP staining to assess the femoral histomorphometry in order to investigate the impact of nodakenin on estrogen shortage-induced bone loss. The findings demonstrated that a decrease in estrogen levels led to a notable rise in osteoclasts, while nodakenin exhibited the ability to impede osteoclastogenesis in mice (Figure 2E,F).

### 2.3. Nodakenin Alters OVX-Induced Gut Microbiota Imbalance in Mice

**Figure 3:**
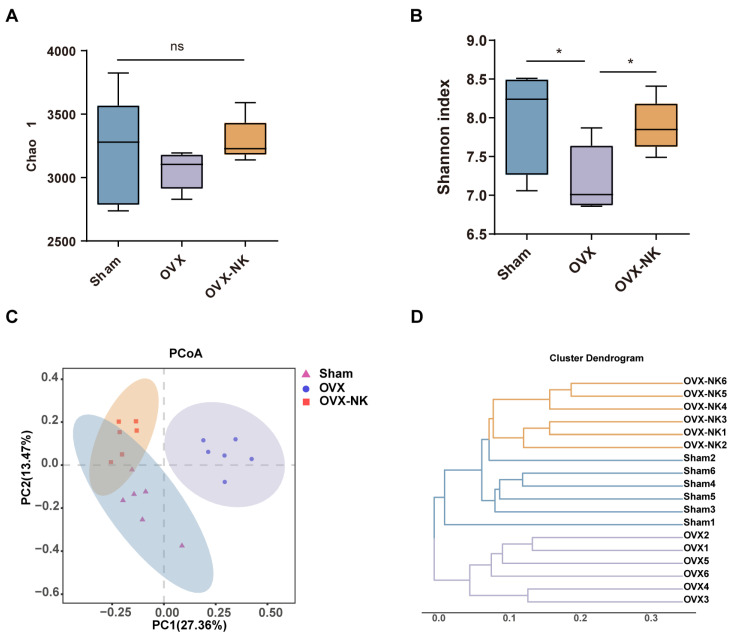
Clustering and diversity analysis of gut microbiota in different groups of mice. Changes in fecal α-diversity of gut microorganisms: Chao 1 estimator (**A**) and Shannon index (**B**). (**C**) Plot-based UniFrac PCoA. (**D**) Cluster analysis of gut microbiota among samples. Mean ± SEM (*n* = 6). * *p* < 0.05, ns means no significant difference.

Recent research has demonstrated that the gut microbiota significantly influences bone metabolism [25,26]. Therefore, we analyzed the composition of the gut microbiota via the systematic sequencing of 16S rRNA gene amplicons in fecal samples. First, the Sobs and Shannon indices flattened the dilution curves, suggesting that the sequencing quantities could adequately capture the majority of the microbial diversity present in the samples (Supplementary Figure S2). Chao 1 analysis showed no significant differences in the microbial community richness between groups (Figure 3A). The Shannon index showed an increased diversity of gut microbial species after nodakenin treatment compared to OVX mice (Figure 3B). Both UniFrac-based principal coordinates analysis (PCoA) and hierarchical clustering showed significant differences in the gut microbiota of OVX mice compared to nodakenin-treated mice (Figure 3C,D). The findings indicate that nodakenin may ameliorate OVX-induced osteoporosis by regulating the imbalance of the gut microbiota.

**Figure 4:**
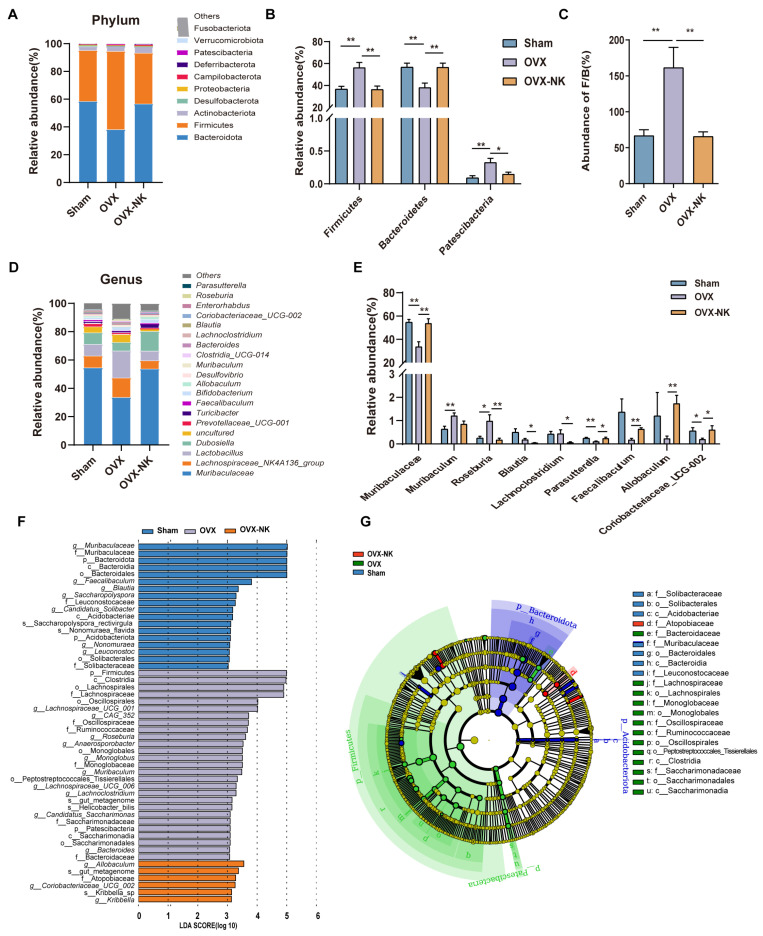
Relative abundance analysis of the gut microbiota at the multispecies level for different groups. (**A**) Composition of gut microbiota at the phylum level. (**B**) Gut microbiota are significantly different at the phylum level. (**C**) Abundance ratio of F/B. (**D**) Composition of gut microbiota at the phylum level. (**E**) Gut microbiota significantly differ at the genus level. (**F**) Histogram of the distribution of LDA values. (**G**) Evolutionary branching diagram of LEfSe analysis. Mean ± SEM (*n* = 6). * *p* < 0.05; ** *p* < 0.01.

To analyze particular alterations in the gut microbiota, we assessed the relative abundance of the top 10 or 20 major taxa. At the phylum level, Bacteroides and Firmicutes were the most dominant phyla, accounting for more than 90% of the total microbial composition (Figure 4A). The research showed that there was a higher proportion of Firmicutes and Patescibacteria and a lower proportion of Bacteroidetes in OVX mice compared to the Sham mice. Nodakenin treatment decreased the relative abundance of Firmicutes and Patescibacteria and increased the relative abundance of Bacteroidetes (Figure 4B). In addition, the ratio of Firmicutes and Bacteroidetes (F/B) is often used as an assessment of the impact of the gut microbe on numerous disease species. Our study found a notable rise in the F/B ratio in OVX mice compared to the Sham mice, while nodakenin treatment decreased the F/B ratio (Figure 4C). At the genus level, the relative abundances of *Muribaculaceae*, *Parasutterella*, and *Coriobacteriaceae_UCG-002* were significantly reduced, and the relative abundances of *Muribaculum* and *Roseburia* were increased in OVX mice. Meanwhile, nodakenin treatment restored the relative abundances of *Muribaculaceae*, *Parasutterella*, *Faecalibaculum*, *Allobaculum*, and *Coriobacteriaceae_UCG-002*, and decreased the relative abundances of *Roseburia*, *Blautia*, and *Lachnoclostridium* (Figure 4D,E). Additionally, to find bacterial taxonomic markers linked to OVX-induced OP, we conducted a further LEfSe analysis (Figure 4F) and created branching diagrams (Figure 4G). Significant differences in bacterial taxonomic composition were observed in different groups. In the nodakenin group, *g__Allobaculum*, *s__gut_metagenome*, f__Atopobiaceae, *g__Coriobacteriaceae_UCG_002*, *s__Kribbella_sp*, and *g__Kribbella* were identified (Figure 4G). Taken together, our data suggest that nodakenin treatment modulates OVX-induced GM imbalance in OP mice.

**Figure 5:**
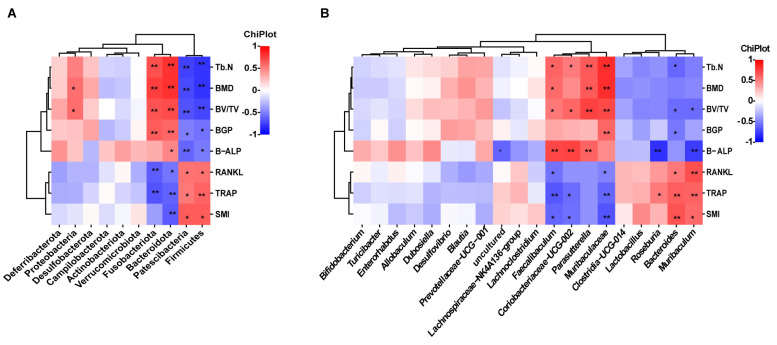
Analysis of correlations between gut microbiota and bone turnover indicators. (**A**) Heat map of Spearman’s r correlation between gut microbiota and bone biological parameters at the phylum level. (**B**) Heat map of Spearman’s r correlation between gut microbiota and bone biological parameters at the genus level.* *p* < 0.05; ** *p* < 0.01.

Spearman correlation analysis was used to evaluate the associations between alterations in the gut microbiota composition and the markers associated with osteoporosis. At the phylum level, Patescibacteria and Firmicutes exhibited a positive correlation with RANKL, TRAP, and SMI, while displaying a negative correlation with Tb.N, BMD, BV/TV, BGP, and B-ALP. Bacteroidota showed a positive correlation with Tb.N, BMD, BV/TV, BGP, and B-ALP and a negative correlation with RANKL, TRAP, and SMI. Fusobacteriota showed a positive association with Tb.N, BMD, BV/TV, and BGP, while it exhibited a negative association with RANKL and TRAP. Proteobacteria showed a positive correlation with BMD and BV/TV (Figure 5A). At the genus level, *Muribaculaceae*, *Faecalibaculum*, and *Coriobacteriaceae_UCG-002* were positively correlated with Tb.N, BMD, BV/TV, and bone formation-associated BTMs, and negatively correlated with TRAP, SMI, and RANKL. *Parasutterella* was positively correlated with Tb.N, BMD, BV/TV, and B-ALP. *Muribaculum* and *Bacteroides* were positively correlated with RANKL, TRAP, and SMI. *Muribaculum* was negatively correlated with BV/TV and B-ALP. *Bacteroides* was negatively correlated with BV/TV, Tb.N, and BGP. *Roseburia* was negatively correlated with B-ALP and positively correlated with TRAP (Figure 5B). *Muribaculum* was negatively correlated with BV/TV and B-ALP. *Bacteroides* had a negative correlation with BV/TV, Tb.N, and BGP. *Roseburia* had a negative correlation with B-ALP and a substantial positive correlation with TRAP (Figure 5B).

### 2.4. Metabolite Profiles Change Caused by Nodakenin Treatment

**Figure 6:**
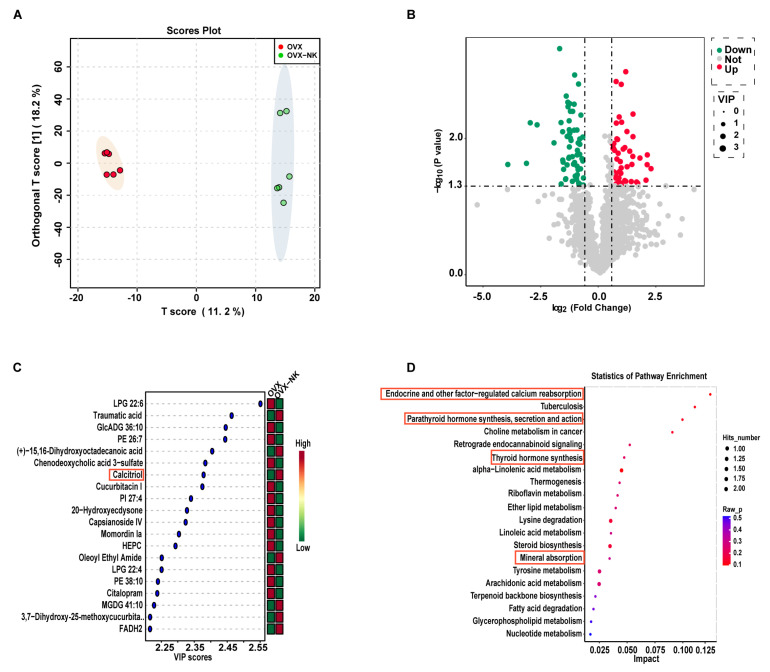
Composition and differences in gut metabolites and analysis of metabolic pathways. (**A**) OPLS-DA analysis of the OVX group versus the nodakenin-treated group. (**B**) Volcano plot of differential metabolites. (**C**) VIP value plot of differential metabolites (top 20 VIP values). (**D**) KEGG differential metabolic pathway plot (top 20 metabolic pathway hits).

Alterations in the composition of gut microbes can result in changes in intestinal metabolites. To further explore the effect of nodakenin on intestinal metabolites, an untargeted metabolomic analysis of feces was performed using LC-MS. The PLS-DA analysis showed that the sample points in the OVX and OVX-NK groups did not overlap at all. This means that the mixtures of metabolites were very different (Figure 6A). The variable significances of VIP > 1 and *p* < 0.05 were used to screen for differential metabolites between the two groups. Out of a total of 2080 metabolites, 113 showed significant differences. Among them, 48 exhibited considerably higher relative levels, while 65 had significantly lower relative levels (Figure 6B). The top 20 differential metabolites with VIP values were selected for presentation, including LPG 22:6, traumatic acid, and calcitriol (Figure 6C). Notably, the group that was administered nodakenin had much higher levels of calcitriol, a metabolite that helps bones form (Figure 6C). Further metabolite enrichment analysis showed that nodakenin treatment had significant effects on multiple metabolic pathways, involving endocrine and other factor-regulated calcium reabsorption, tuberculosis, retrograde endocannabinoid signaling, alpha-Linolenic acid metabolism, thermogenesis, riboflavin metabolism, ether lipid metabolism, mineral absorption, etc., (Figure 6D).

**Figure 7:**
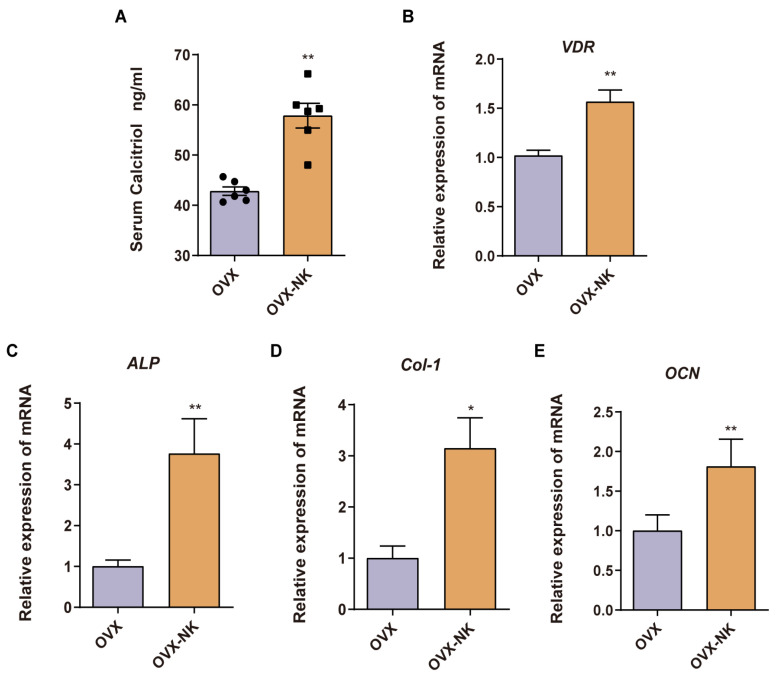
Nodakenin prevents OP by accumulating calcitriol. (**A**) ELISA detection of calcitriol in mice serum. (**B**–**E**) qRT-PCR was performed to determine the levels of *VDR*, *ALP*, *Col-1* and *OCN* mRNA expression. mean ± sem (*n* = 6). * *p* < 0.05, ** *p* < 0.01.

Consistent with our metabolomics findings, we observed a notable increase in serum levels of calcitriol, a pivotal metabolite, in the nodakenin-treated group compared to OVX mice (Figure 7A). Calcitriol functions by binding to the vitamin D receptor (VDR). Remarkably, we found a significant elevation in *VDR* expression within the bone tissue of nodakenin-treated mice (Figure 7B). Furthermore, the mRNA levels of key osteogenic marker genes, including alkaline phosphatase (*ALP*), collagen type I (*Col-1*), and osteocalcin (*OCN*), exhibited significant increases (Figure 7C–E).

**Figure 8:**
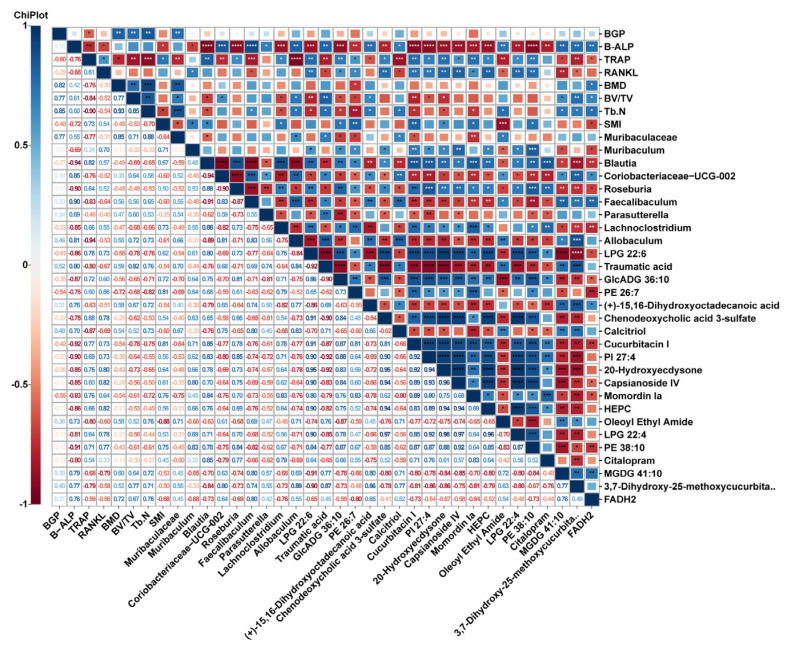
Heatmap of Spearman-analyzed bone-related metrics, gut microbiota, and top 20 metabolites. * *p* < 0.05, ** *p* < 0.01, *** *p* < 0.001 and *** *p* < 0.0001.

In addition, Spearman correlation analysis was performed on intestinal metabolites (VIP top 20), significantly different gut microbiota, and osteoporosis indicators (Figure 8). We discovered substantial associations among the aforementioned metrics, indicating that the beneficial impact of nodakenin on bone loss in mice produced by OVX involves the regulation and recovery of gut microbiota and metabolite profiles. Nevertheless, additional verification is required to establish the potential pathways that influence the gut microbiota and metabolites in OP therapy.

### 2.5. Nodakenin Ameliorates Intestinal Barrier Impairment in OVX Mice

**Figure 9:**
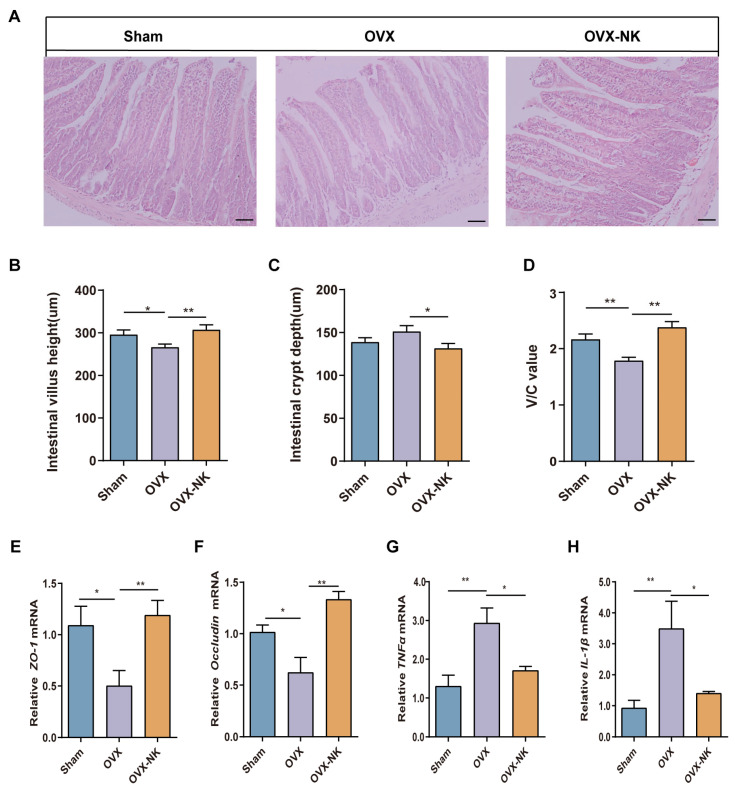
Nodakenin ameliorates intestinal barrier impairment in OVX mice. (**A**) Representative images of hematoxylin and eosin staining. Scale bars are 100 μm. (**B**–**D**) Image J measured the height of small intestinal villi and depth of crypts and analyzed the V/C ratio. (**E**–**H**) qRT-PCR was performed to determine the levels of *Occludin*, *ZO-1*, *IL-1β* and *TNFα* mRNA expression. mean ± sem (*n* = 6). * *p* < 0.05, ** *p* < 0.01.

To explore the effect of nodakenin on intestinal barrier integrity in OVX-induced OP mice, we analyzed the intestinal barrier via an intestinal villus morphology assessment (Figure 9A). Compared with that in the Sham group, the height of the intestinal villi was decreased in OVX mice (Figure 9B), while the depth of intestinal crypts was increased (Figure 9C) and the V/C ratio (Figure 9D) was significantly decreased. It was also found that the OVX mice had much lower levels of intestinal *Occludin* (Figure 9E) and *Zonulae-1* (*ZO-1*) (Figure 9F) mRNA, while they had much higher levels of *IL-1β* and *TNFα* mRNA (Figure 9G,H). Nodakenin treatment elevated the intestinal villus height, and *Occludin* and *ZO-1* mRNA levels, and decreased the depth of crypts and the expression of inflammatory factors in the intestines of OVX mice (Figure 9B–H). It is suggested that nodakenin can improve OVX-induced intestinal barrier impairment.

## 3. Discussion

The burden of OP and OP-induced fractures is increasing with global population aging [27]. In the present study, our data demonstrated that nodakenin treatment elevated bone mass, modulated serum bone turnover indexes, and decreased osteoclast activity. Generally, the glycosides contained in herbal medicines are poorly absorbed in the intestine. Due to their low bioavailability, glycosides are retained in the intestine for a considerable period. After being metabolized by the gut microbiota, they are mainly absorbed in the form of glycosides, although it has been shown that the oral bioavailability of nodakenin is extremely low [23], which greatly limits its absorption. However, the oral administration of nodakenin still significantly elevated bone mineral density in OVX mice. It is suggested that it may exert its pharmacological effects by affecting the gut microbiota and the produced metabolites. There is increasing evidence that GM imbalance is associated with the pathogenesis and clinical manifestations of OP [28]. Therefore, the present study focused on the possible mechanism of action of nodakenin in suppressing OP in terms of the gut/bone axis.

The gastrointestinal tract is acknowledged as a distinct organ system that has a significant impact on the maintenance of bone health. Recent research has indicated that there are connections between the stomach, bone signaling pathways, and microbiota that play a role in the regulation of bone health [29,30,31,32]. Firmicutes can activate osteoclasts and exacerbate inflammation [33]. The abundance and proportion (F/B ratio) of Firmicutes and Bacteroides are important factors indicating gut microbiota disorders, which may reflect the process of disease development. In this study, Firmicutes and Bacteroides were the two dominant species, accounting for more than 90% of the gut microbiota at the phylum level. In the OVX group, the relative abundance of Firmicutes was significantly increased and significantly negatively correlated with BMD, BV/TV, etc., and the F/B ratio was significantly increased. The increase in the abundance of Firmicutes and the increase in the F/B ratio were reversed after nodakenin treatment. Consistent with the present study, it has been found that the ratio of Firmicutes to Bacteroides was negatively correlated with bone volume [34,35]. Furthermore, Bacteroides was significantly reduced in OVX mice and nodakenin treatment restored its relative abundance. A positive correlation between Bacteroides and calcium uptake has been reported [36]. At the genus level, nodakenin treatment restored OVX-induced reductions in the relative abundances of *Muribaculaceae*, *Parasutterella*, *Faecalibaculum*, *Allobaculum*, and *Coriobacteriaceae_UCG-002* compared to the OVX group. *Muribaculaceae* and *Parasutterella* were positively correlated with BMD, BV/TV, Tb.N, and B-ALP. Palmatine and intermittent parathyroid hormone (PTH) have been reported to increase the abundance of *Muribaculaceae* and increase bone mass in OVX rats [37,38]. *Parasutterella* is involved in Kefir Peptides’ prevention of estrogen deficiency-induced bone loss and GM disorders [13]. *Allobaculum*, a known butyrate producer is involved in the anti-osteoporotic effects of several active compounds [39,40]. *Faecalibaculum* and *Coriobacteriaceae_UCG-002*, although not yet reported to be associated with increased bone volume, play an important role in the maintenance of intestinal homeostasis and in preventing intestinal damage [41,42,43]. In conclusion, these flora may be involved in the correction of nodakenin anti-OVX-induced GM ecological imbalances.

Metabolomics is a technique used to investigate and analyze small-molecule metabolites in order to understand and describe pathological alterations. The use of metabolomics allows for the screening of metabolic biomarkers, which can help to elucidate the pathogenic mechanisms of osteoporosis and develop new drug targets [44]. Our study showed that nodakenin treatment significantly altered the fecal metabolite profile in OVX mice. During nodakenin alleviation of OP, metabolomic screening yielded a significant upregulation of calcitriol, a differential metabolite closely related to osteoporosis, and four signaling pathways associated with calcitriol were identified by KEGG enrichment analysis. Therefore, calcitriol might be a key metabolite regulated by nodakenin. Calcitriol has been reported as a drug used in the clinical treatment of osteoporosis, and it is also one of the most important active metabolites of vitamin D3, which enhances intestinal calcium absorption [45,46]. It is also a potent transcriptional activator of osteogenesis-related genes encoding type I collagen (Col-I), alkaline phosphatase (ALP), and osteocalcin (OCN) in osteoblasts, which regulate calcium homeostasis through stimulating osteoblast activity, thereby combating osteoporosis [47,48]. Similarly, a significant increase in calcitriol was detected in the fecal metabolites of nodakenin-treated mice. Also, a significant increase in the levels of calcitriol was confirmed in the serum of nodakenin-treated mice, which was accompanied by an increase in the expression of the osteogenic marker genes ALP, OCN, and Col-1 in bone tissue. This suggests that calcitriol is a key metabolite of nodakenin in alleviating OP. In addition, both epidemiological investigations and basic studies have shown that there is a link between obesity and osteoporosis, with multiple important and intersecting molecular and cellular signaling pathways [49]. Estrogen deficiency-induced weight gain has also been induced in mouse models of osteoporosis [50]. LPG 22:6 has been found to be significantly elevated in high-fat-induced obese mice [51]. Our study showed that LPG 22:6 was elevated in OVX mice, whereas nodakenin treatment decreased the level of LPG 22:6. Traumatic acid is a potent wound-healing agent in plants with antioxidant properties. It can also control lipid accumulation in adipocytes and reduce obesity caused by a high-fat diet [52]. Our study showed that traumatic acid was decreased in OVX mice and increased under nodakenin treatment. It is suggested that the alteration in these obesity-related metabolites may be the key to nodakenin’s ability to inhibit OVX-induced weight gain. In addition, KEGG enrichment analysis revealed that of the pathways in which nodakenin alleviated the significant changes induced by OP, four of them were closely associated with osteoporosis, including endocrine and other factor-regulated calcium reabsorption; parathyroid hormone synthesis, secretion, and action; thyroid hormone synthesis and mineral absorption. Moreover, calcitriol, as a hub of metabolites is closely related to these four pathways. It plays a crucial role in bone regeneration by binding to the vitamin D receptor (VDR) to activate various downstream transcription factors involved in signaling pathways such as calcium reabsorption, hormone synthesis and secretion [53,54,55,56]. Consistent with this, our experiments showed that VDR expression was elevated in the bone tissue of nodakenin-treated mice.

Estrogen deficiency is tightly linked to the disruption of the intestinal mucosal barrier, which adversely affects skeletal properties through the modulation of the GM-bone axis [57,58]. PMOP is strongly associated with systemic chronic inflammation, which is often accompanied by the augmentation of pro-osteoclastogenic factors such as TNF-α and IL-1β [59,60]. Interestingly, nodakenin-treated mice exhibit reduced levels of expression of the pro-osteoclastogenic factors TNF-α and IL-1β in the intestine, which is likely an important reason for their inhibition of osteoclast formation. Increased intestinal permeability has been shown to play a key role in the regulation of inflammation and is inextricably linked to the colonization of Firmicutes as well as the diversity of gut microbes [33]. Our study provides strong support for this view, as nondakenin treatment significantly reduced intestinal permeability in OVX mice, accompanied by a decrease in the release of pro-inflammatory cytokines IL-1β and TNF-α. This phenomenon is likely related to the role of nondakenin in enhancing gut microbial diversity and reducing the relative abundance of Firmicutes. Notably, calcitriol protects the barrier function of endothelial and epithelial cells and effectively inhibits intestinal inflammation [61]. In our study, nodakenin-treated mice exhibited a significant rise in calcitriol, suggesting that the reduction in intestinal permeability and expression of pro-inflammatory factors by nodakenin may be related to calcitriol. This finding not only deepens our understanding of the role of nodakenin in maintaining intestinal health but also provides new ideas for future therapies targeting bone-related diseases.

## 4. Materials and Methods

### 4.1. Animal Experiment and Design

We used C57BL/6J mice (6–8 weeks); one week after acclimatization, six mice were randomly selected as controls (Sham) for cesarean section and the rest of the mice received bilateral ovariectomy. In detail, after anesthesia with 1% sodium pentobarbital, the dorsal test fur was shaved and disinfected, and then, the bilateral ovaries were removed and the oviducts were ligated. After surgery, OVX mice were randomly divided into two groups (*n* = 6 in each group) and the vehicle (intragastric administration) (1% DMSO + 5% tween 80 + saline) and nodakenin (Chemfaces, Wuhan, China) (20 mg/kg nodakenin + 5% tween 80 + saline) were administered once daily for 6 weeks. Body weights were measured at the end of the experiment, and serum, feces, femurs, and viscera were collected for subsequent experimental analysis. The Qingdao Agricultural University granted approval for all the animal experimentation processes.

### 4.2. Micro-CT (μCT) Analysis

Mouse femurs were dissected and then analyzed using a SkyScan-1276 X-ray microtomograph (Micro-CT) (Bruker, Billerica, MA, Germany). Using the bottom-most portion of the lateral growth plate of the femoral knee as the baseline, the region of the bone marrow cavity, with a thickness of 1 mm underneath was selected and designated as the region of interest (ROI) for 3D reconstruction; 3D images were reconstructed using N-Recon software and analyzed in three dimensions using CT-AN software.

### 4.3. Determination of Biochemical Indicators

Enzyme-linked immunosorbent assay (ELISA) kits were used to detect the serum levels of bone turnover markers (BTMs), including alkaline phosphatase (B-ALP), osteocalcin (BGP), tartrate-resistant acid phosphatase (TRAP), receptor activator of nuclear factor-κB ligand (RANKL) and Calcitriol (Jiangsu Meimian Industrial Co., Ltd., Yancheng, China).

### 4.4. Histomorphometric Analysis

The femurs of mice, with the exception of muscle tissue were immersed in a 4% paraformaldehyde solution for 24 h. Subsequently, they were transferred to an EDTA decalcification solution for an additional 4 weeks. Paraffin-embedded sections were cut into 4 µm thick sections and stained with TRAP. The sections were observed under a microscope, and images were captured.

Sections of paraffin-embedded colon tissue (5 µm) were stained with hematoxylin and eosin to assess intestinal barrier integrity. The morphological characteristics of intestinal villi, including the intestinal villus height (µm), intestinal crypt depth (µm), and the ratio of villus height to crypt depth (V/C) were assessed using Image-Pro Plus.

### 4.5. Quantitative Real-Time PCR

Mouse colon RNA was prepared by using TriZol reagent (Invitrogen, Waltham, MA, USA) and then quantified using a Nano Drop 2000 (Thermo Fisher, Waltham, MA, USA). The reverse transcription of cDNA was performed using the PrimeScript RT Reagent Kit with gDNA Eraser (Takara, Kyoto, Japan). A real-time polymerization chain reaction was performed using a CFX96 Real-Time Fluorescence Quantitative PCR System (Bio-Rad, Hercules, CA, USA). GAPDH was used as an internal reference, and the primers used are shown in Supplementary Table S1. The 2^−ΔΔCT^ method was used for analysis.

### 4.6. 16S rRNA Analysis

Fresh mouse feces were collected and frozen at −80 °C (*n* = 6). Total genomic DNA was extracted from the fecal samples and assayed for quality using a Nanodrop 2000 and gel electrophoresis. 16rRNA gene V3-V4 region amplification was performed using the Bacterial Universal Priming Kit (Omega Bio-tek, Norcross, GA, USA), and paired-end sequencing (2 × 250 bp) was carried out with the Illumina MiSeq platform (Illumina, San Diego, CA, USA) by Beijing Allwegene Tech, Ltd. (Beijing, China). Based on the OTUs and richness results, the Alpha Diversity Index and Beta Diversity Distance Matrix were calculated using QIIME v1.8.0 software [62]. The Bray–Curtis distance was measured using the R package (R version 3.6.0) to compare the differences in microbial composition between groups. Linear discriminant analysis (LDA), combined with effect size (LEfSe, Blair, WI, USA, Galaxy version 1.0) was used to identify key OTUs that responded to different interventions.

### 4.7. Metabolic Analysis of Feces by LC-MS/MS

The samples were removed from the freezer at −80 °C and thawed slowly at 4 °C. An appropriate amount of feces (*n* = 6) was added into pre-cooled MeOH:ACN:H_2_O (*v*:*v*:*v* = 2:2:1) solution and ground and sonicated in order to homogenize the sample; the sample was allowed to stand at −20 °C for 1 h and then centrifuged at 13,000 rpm for 15 min at 4 °C. The supernatant was then freeze-dried. For mass spectrometry analysis, an appropriate amount of ACN:H_2_O solution (*v*:*v* = 1:1) was added for vortexing and sonication to make it fully re-dissolve, and then centrifuged at 13,000 rpm 4 °C for 15 min; the supernatant was aspirated into the injection bottle and then analyzed by LC-MS/MS.

Metabolomics was used for separation using a Waters ACQUITY UPLC HSS T3 column (2.1 mm × 100 mm, 1.8 μm, Milford, MA, USA). The mobile phases were A (0.1% formic acid in water) and B (0.1% formic acid in acetonitrile) for gradient elution: 95% B, 0.5 min; 95–75% B, 0.5–2.5 min; 70–100% B, 2.5–7.5 min; 100% B, 7.5–9 min; 100–5% B, 9–9.5 min and 5% B, 9–12 min. The injection volume was 2 μL, the flow rate was 0.3 mL/min, and the column temperature was 50 °C. The sample was collected in the positive and negative ion modes. Sample metabolic analytes exiting the column were collected once in positive ion mode and once in negative ion mode using a high-resolution mass spectrometer, Triple TOF 5600+(SCIEX, Washington, DC, USA). The details of the parameters are as follows: Ion Source Gas: 150 psi; Ion Source Gas2: 50 psi; Curtain Gas: 35 psi; Source Temperature: 500 °C; IonSapary Voltage Floating:5500 V and −4500 V (positive and negative); Declustering Potential (DP): ±80 V (positive and negative); TOF MS scan *m*/*z* range:60–1200 Da; Product Ion Scan *m*/*z* Range: 25–1200 Da; TOF MS Scan Accumulation Time: 0.25 s/spectra; Product Ion Scan Accumulation Time: 0.03 s/spectra. The secondary mass spectra were obtained by Information Dependent Acquisition (IDA), and the extracted data were analyzed for metabolite structure identification and data analysis using the High Sensitivity mode, CE: 30 V ± 15, to screen for different metabolites in each group.

Peak identification, peak extraction, peak alignment, and integration were performed using an R package written in-house (kernel XCMS), followed by metabolite identification and screening using the in-house MS2 database (Allwegene, Beijing, China). The differential metabolic pathways were screened against authoritative metabolite databases such as KEGG and PubChem.

### 4.8. Statistics

All data are presented as the mean ± standard deviation. *t*-tests were employed for comparisons between two groups, and one-way ANOVA was used for multiple comparisons. Spearman’s correlation analysis was used to analyze the relationship between the gut microbiota, differential metabolites, and bone metabolic parameters. Heat map correlation statistics were analyzed using SPSS 25.0 (IBM). * *p* < 0.05; ** *p* < 0.01.

## 5. Conclusions

In summary, the present study confirmed that nodakenin modulates gut microbiota composition and gut metabolite disorders and restores intestinal permeability. Nodakenin treatment decreased the F/B ratio, as well as produced some specific gut microbiota changes at the genus level. Meanwhile, nodakenin may affect hormone synthesis and calcium reabsorption by regulating metabolites such as calcitriol, thereby ameliorating osteoporosis. The present study provides new perspectives for exploring nodakenin’s anti-osteoporotic effects and lays the foundation for considering gut microbiota as a drug target.

## Data Availability

All data generated or analyzed during this study will be available from the corresponding author on reasonable request. The data are not publicly available due to confidentiality required to continue the study.

## Supplementary Materials

The following supporting information can be downloaded at: https://www.mdpi.com/article/10.3390/molecules29061240/s1, Figure S1: Body weight and organ weights; Figure S2: Rarefaction curves and Shannon-Wiener curves; Table S1: Primer sequence for qRT-PCR.

## References

[1] Walker M.D., Shane E. (2023). Postmenopausal Osteoporosis. N. Engl. J. Med..

[2] Ishikawa K., Nagai T., Sakamoto K., Ohara K., Eguro T., Ito H., Toyoshima Y., Kokaze A., Toyone T., Inagaki K. (2016). High bone turnover elevates the risk of denosumab-induced hypocalcemia in women with postmenopausal osteoporosis. Ther. Clin. Risk Manag..

[3] Zhang Y.W., Cao M.M., Li Y.J., Dai G.C., Lu P.P., Zhang M., Bai L.Y., Chen X.X., Shi L., Zhang C. (2022). Dietary Protein Intake in Relation to the Risk of Osteoporosis in Middle-Aged and Older Individuals: A Cross-Sectional Study. J. Nutr. Health Aging.

[4] Zhu Y., Huang Z., Wang Y., Xu W., Chen H., Xu J., Luo S., Zhang Y., Zhao D., Hu J. (2020). The efficacy and safety of denosumab in postmenopausal women with osteoporosis previously treated with bisphosphonates: A review. J. Orthop. Transl..

[5] Arceo-Mendoza R.M., Camacho P.M. (2021). Postmenopausal Osteoporosis: Latest Guidelines. Endocrinol. Metab. Clin. N. Am..

[6] Ke X., Walker A., Haange S.-B., Lagkouvardos I., Liu Y., Schmitt-Kopplin P., von Bergen M., Jehmlich N., He X., Clavel T. (2019). Synbiotic-driven improvement of metabolic disturbances is associated with changes in the gut microbiome in diet-induced obese mice. Mol. Metab..

[7] Ejtahed H.S., Soroush A.R., Angoorani P., Larijani B., Hasani-Ranjbar S. (2016). Gut Microbiota as a Target in the Pathogenesis of Metabolic Disorders: A New Approach to Novel Therapeutic Agents. Horm. Metab. Res..

[8] John G.K., Mullin G.E. (2016). The Gut Microbiome and Obesity. Curr. Oncol. Rep..

[9] Zheng P., Li Z., Zhou Z. (2018). Gut microbiome in type 1 diabetes: A comprehensive review. Diabetes Metab. Res. Rev..

[10] Cheng S., Qi X., Ma M., Zhang L., Cheng B., Liang C., Liu L., Li P., Kafle O.P., Wen Y. (2020). Assessing the Relationship Between Gut Microbiota and Bone Mineral Density. Front. Genet..

[11] Zhou T., Wang M., Ma H., Li X., Heianza Y., Qi L. (2021). Dietary Fiber, Genetic Variations of Gut Microbiota-derived Short-chain Fatty Acids, and Bone Health in UK Biobank. J. Clin. Endocrinol. Metab..

[12] Palacios-González B., Ramírez-Salazar E.G., Rivera-Paredez B., Quiterio M., Flores Y.N., Macias-Kauffer L., Moran-Ramos S., Denova-Gutiérrez E., Ibarra-González I., Vela-Amieva M. (2020). A Multi-Omic Analysis for Low Bone Mineral Density in Postmenopausal Women Suggests a RELATIONSHIP between Diet, Metabolites, and Microbiota. Microorganisms.

[13] Tu M.-Y., Han K.-Y., Chang G.R.-L., Lai G.-D., Chang K.-Y., Chen C.-F., Lai J.-C., Lai C.-Y., Chen H.-L., Chen C.-M. (2020). Kefir Peptides Prevent Estrogen Deficiency-Induced Bone Loss and Modulate the Structure of the Gut Microbiota in Ovariectomized Mice. Nutrients.

[14] Chen X., Zhang Z., Hu Y., Cui J., Zhi X., Li X., Jiang H., Wang Y., Gu Z., Qiu Z. (2020). Lactulose Suppresses Osteoclastogenesis and Ameliorates Estrogen Deficiency-Induced Bone Loss in Mice. Aging Dis..

[15] Li B., Liu M., Wang Y., Gong S., Yao W., Li W., Gao H., Wei M. (2020). Puerarin improves the bone micro-environment to inhibit OVX-induced osteoporosis via modulating SCFAs released by the gut microbiota and repairing intestinal mucosal integrity. Biomed. Pharmacother..

[16] Lee N.Y., Chung K.-S., Jin J.S., Lee Y.-C., An H.-J. (2017). The Inhibitory Effect of Nodakenin on Mast-Cell-Mediated Allergic Inflammation Via Downregulation of NF-κB and Caspase-1 Activation. J. Cell Biochem..

[17] Lim J.-Y., Lee J.-H., Yun D.-H., Lee Y.-M., Kim D.-K. (2021). Inhibitory effects of nodakenin on inflammation and cell death in lipopolysaccharide-induced liver injury mice. Phytomedicine.

[18] Li J., Wang L., Tan R., Zhao S., Zhong X., Wang L. (2020). Nodakenin alleviated obstructive nephropathy through blunting Snail1 induced fibrosis. J. Cell. Mol. Med..

[19] Jin B.-R., Lee M., An H.-J. (2021). Nodakenin represses obesity and its complications via the inhibition of the VLDLR signalling pathway in vivo and in vitro. Cell Prolif..

[20] Kim T.W. (2023). Nodakenin Induces ROS-Dependent Apoptotic Cell Death and ER Stress in Radioresistant Breast Cancer. Antioxidants.

[21] Yi N., Mi Y., Xu X., Li N., Chen B., Yan K., Tan K., Zhang B., Wang L., Kuang G. (2022). Nodakenin attenuates cartilage degradation and inflammatory responses in a mice model of knee osteoarthritis by regulating mitochondrial Drp1/ROS/NLRP3 axis. Int. Immunopharmacol..

[22] Liu C., Zhao M., Chen J., Xu L., Wang K., Li G. (2023). Nodakenin alleviates ovariectomy-induced osteoporosis by modulating osteoblastogenesis and osteoclastogenesis. Eur. J. Pharmacol..

[23] Zhang P., Yang X.-W. (2009). Biotransformation of nodakenin and simultaneous quantification of nodakenin and its aglycone in incubated system of human intestinal bacteria by HPLC method. J. Asian Nat. Prod. Res..

[24] Feng W., Ao H., Peng C., Yan D. (2019). Gut microbiota, a new frontier to understand traditional Chinese medicines. Pharmacol. Res..

[25] Kang D.D., Li F., Kirton E., Thomas A., Egan R., An H., Wang Z. (2019). MetaBAT 2: An adaptive binning algorithm for robust and efficient genome reconstruction from metagenome assemblies. PeerJ.

[26] Ohlsson C., Sjögren K. (2018). Osteomicrobiology: A New Cross-Disciplinary Research Field. Calcif. Tissue Int..

[27] Reid I.R. (2020). A broader strategy for osteoporosis interventions. Nat. Rev. Endocrinol..

[28] Behera J., Ison J., Tyagi S.C., Tyagi N. (2020). The role of gut microbiota in bone homeostasis. Bone.

[29] Britton R.A., Irwin R., Quach D., Schaefer L., Zhang J., Lee T., Parameswaran N., McCabe L.R., Probiotic L. (2014). reuteri treatment prevents bone loss in a menopausal ovariectomized mouse model. J. Cell Physiol..

[30] Chen D., Zhao C.-M. (2011). The possible existence of a gut-bone axis suggested by studies of genetically manipulated mouse models?. Curr. Pharm. Des..

[31] Hao M.-L., Wang G.-Y., Zuo X.-Q., Qu C.-J., Yao B.-C., Wang D.-L. (2019). Gut microbiota: An overlooked factor that plays a significant role in osteoporosis. J. Int. Med. Res..

[32] Sjögren K., Engdahl C., Henning P., Lerner U.H., Tremaroli V., Lagerquist M.K., Bäckhed F., Ohlsson C. (2012). The gut microbiota regulates bone mass in mice. J. Bone Miner. Res..

[33] Yatsonsky Ii D., Pan K., Shendge V.B., Liu J., Ebraheim N.A. (2019). Linkage of microbiota and osteoporosis: A mini literature review. World J. Orthop..

[34] Li C., Huang Q., Yang R., Dai Y., Zeng Y., Tao L., Li X., Zeng J., Wang Q. (2019). Gut microbiota composition and bone mineral loss-epidemiologic evidence from individuals in Wuhan, China. Osteoporos. Int..

[35] Guo M., Liu H., Yu Y., Zhu X., Xie H., Wei C., Mei C., Shi Y., Zhou N., Qin K. (2023). Lactobacillus rhamnosus GG ameliorates osteoporosis in ovariectomized rats by regulating the Th17/Treg balance and gut microbiota structure. Gut Microbes.

[36] Weaver C.M. (2015). Diet, gut microbiome, and bone health. Curr. Osteoporos. Rep..

[37] Jie L., Ma Z., Gao Y., Shi X., Yu L., Mao J., Wang P. (2023). The mechanism of palmatine-mediated intestinal flora and host metabolism intervention in OA-OP comorbidity rats. Front. Med..

[38] Zhou J., Wang R., Zhao R., Guo X., Gou P., Bai H., Lei P., Xue Y. (2022). Intermittent Parathyroid Hormone Alters Gut Microbiota in Ovariectomized Osteoporotic Rats. Orthop. Surg..

[39] Zhou J., Cheng J., Liu L., Luo J., Peng X. (2023). Lactobacillus acidophilus (LA) Fermenting Astragalus Polysaccharides (APS) Improves Calcium Absorption and Osteoporosis by Altering Gut Microbiota. Foods.

[40] He W., Xie Z., Thøgersen R., Rasmussen M.K., Zachariassen L.F., Jørgensen N.R., Nørgaard J.V., Andersen H.J., Nielsen D.S., Hansen A.K. (2022). Effects of Calcium Source, Inulin, and Lactose on Gut-Bone Associations in an Ovarierectomized Rat Model. Mol. Nutr. Food Res..

[41] Cao Y.G., Bae S., Villarreal J., Moy M., Chun E., Michaud M., Lang J.K., Glickman J.N., Lobel L., Garrett W.S. (2022). Faecalibaculum rodentium remodels retinoic acid signaling to govern eosinophil-dependent intestinal epithelial homeostasis. Cell Host Microbe.

[42] Zagato E., Pozzi C., Bertocchi A., Schioppa T., Saccheri F., Guglietta S., Fosso B., Melocchi L., Nizzoli G., Troisi J. (2020). Endogenous murine microbiota member Faecalibaculum rodentium and its human homologue protect from intestinal tumour growth. Nat. Microbiol..

[43] Wu J., Guo W., Cui S., Tang X., Zhang Q., Lu W., Jin Y., Zhao J., Mao B., Chen W. (2023). Broccoli seed extract rich in polysaccharides and glucoraphanin ameliorates DSS-induced colitis via intestinal barrier protection and gut microbiota modulation in mice. J. Sci. Food Agric..

[44] Johnson C.H., Ivanisevic J., Siuzdak G. (2016). Metabolomics: Beyond biomarkers and towards mechanisms. Nat. Rev. Mol. Cell Biol..

[45] Tilyard M.W., Spears G.F., Thomson J., Dovey S. (1992). Treatment of postmenopausal osteoporosis with calcitriol or calcium. N. Engl. J. Med..

[46] Thompson L., Wang S., Tawfik O., Templeton K., Tancabelic J., Pinson D., Anderson H.C., Keighley J., Garimella R. (2012). Effect of 25-hydroxyvitamin D3 and 1 α,25 dihydroxyvitamin D3 on differentiation and apoptosis of human osteosarcoma cell lines. J. Orthop. Res..

[47] Tang Q., Hu Z., Jin H., Zheng G., Yu X., Wu G., Liu H., Zhu Z., Xu H., Zhang C. (2019). Microporous polysaccharide multilayer coated BCP composite scaffolds with immobilised calcitriol promote osteoporotic bone regeneration both in vitro and in vivo. Theranostics.

[48] Yang D., Turner A.G., Wijenayaka A.R., Anderson P.H., Morris H.A., Atkins G.J. (2015). 1,25-Dihydroxyvitamin D3 and extracellular calcium promote mineral deposition via NPP1 activity in a mature osteoblast cell line MLO-A5. Mol. Cell. Endocrinol..

[49] Rayalam S., Yang J.-Y., Della-Fera M.A., Baile C.A. (2011). Novel molecular targets for prevention of obesity and osteoporosis. J. Nutr. Biochem..

[50] Mann S.N., Pitel K.S., Nelson-Holte M.H., Iwaniec U.T., Turner R.T., Sathiaseelan R., Kirkland J.L., Schneider A., Morris K.T., Malayannan S. (2020). 17α-Estradiol prevents ovariectomy-mediated obesity and bone loss. Exp. Gerontol..

[51] Chen X., Chen S., Ren Q., Niu S., Yue L., Pan X., Li Z., Zhu R., Jia Z., Chen X. (2022). A metabonomics-based renoprotective mechanism analysis of empagliflozin in obese mice. Biochem. Biophys. Res. Commun..

[52] Gao J., Zhang Z., Dong X., Zhao J., Peng Z., Zhang L., Xu Z., Xu L., Wang X., Guo X. (2023). Traumatic acid inhibits ACSL4 associated lipid accumulation in adipocytes to attenuate high-fat diet-induced obesity. FASEB J..

[53] Watson P.H., Hanley D.A. (1993). Parathyroid hormone: Regulation of synthesis and secretion. Clin. Invest. Med..

[54] Li H., Liu S., Miao C., Lv Y., Hu Y. (2023). Integration of metabolomics and transcriptomics provides insights into enhanced osteogenesis in Ano5Cys360Tyr knock-in mouse model. Front. Endocrinol..

[55] Yang R., Chen J., Zhang J., Qin R., Wang R., Qiu Y., Mao Z., Goltzman D., Miao D. (2020). 1,25-Dihydroxyvitamin D protects against age-related osteoporosis by a novel VDR-Ezh2-p16 signal axis. Aging Cell.

[56] Plum L.A., DeLuca H.F. (2010). Vitamin D, disease and therapeutic opportunities. Nat. Rev. Drug Discov..

[57] Fuhrman B.J., Feigelson H.S., Flores R., Gail M.H., Xu X., Ravel J., Goedert J.J. (2014). Associations of the fecal microbiome with urinary estrogens and estrogen metabolites in postmenopausal women. J. Clin. Endocrinol. Metab..

[58] Flores R., Shi J., Fuhrman B., Xu X., Veenstra T.D., Gail M.H., Gajer P., Ravel J., Goedert J.J. (2012). Fecal microbial determinants of fecal and systemic estrogens and estrogen metabolites: A cross-sectional study. J. Transl. Med..

[59] Adeel S., Singh K., Vydareny K.H., Kumari M., Shah E., Weitzmann M.N., Tangpricha V. (2013). Bone loss in surgically ovariectomized premenopausal women is associated with T lymphocyte activation and thymic hypertrophy. J. Investig. Med..

[60] D’Amelio P., Grimaldi A., Di Bella S., Brianza S.Z.M., Cristofaro M.A., Tamone C., Giribaldi G., Ulliers D., Pescarmona G.P., Isaia G. (2008). Estrogen deficiency increases osteoclastogenesis up-regulating T cells activity: A key mechanism in osteoporosis. Bone.

[61] Chen S., Zhu J., Chen G., Zuo S., Zhang J., Chen Z., Wang X., Li J., Liu Y., Wang P. (2015). 1,25-Dihydroxyvitamin D3 preserves intestinal epithelial barrier function from TNF-α induced injury via suppression of NF-kB p65 mediated MLCK-P-MLC signaling pathway. Biochem. Biophys. Res. Commun..

[62] Caporaso J.G., Kuczynski J., Stombaugh J., Bittinger K., Bushman F.D., Costello E.K., Fierer N., Peña A.G., Goodrich J.K., Gordon J.I. (2010). QIIME allows analysis of high-throughput community sequencing data. Nat. Methods.

