# Supplementary material for: Nodakenin Ameliorates Ovariectomy-Induced Bone Loss by Regulating Gut Microbiota
Source: Molecules. 2024 Mar 11;29(6):1240. doi: 10.3390/molecules29061240 (PMC10976110; doi:10.3390/molecules29061240)
Supplement: Supplementary file 1 [file molecules-29-01240-s001.zip › Supplementary Table S1.pdf]

Table S1. Primer sequence for RT-PCR.

Gene	Primer sequence
Occludin	Forward: 5′- ATGTCCGGCCGATGCTCTC-3′
	Reverse: 5′-TTTGGCTGCTCTTGGGTCTGTAT-3′
ZO-1	Forward: 5′-GCCGCTAAGAGCACAGCAA -3′
	Reverse: 5′-GCCCTCCTTTTAACACATCAGA- 3′
TNFα	Forward: 5′- CAGGCGGTGCCTATGTCTC-3′
	Reverse: 5′-CGATCACCCCGAAGTTCAGTAG-3′
IL-1β	Forward: 5′- GAAATGCCACCTTTTGACAGTG-3′
	Reverse: 5′-TGGATGCTCTCATCAGGACAG-3′
GAPDH	Forward: 5′- AGGTCGGTGTGAACGGATTTG-3′
	Reverse: 5′-GGGGTCGTTGATGGCAACA-3′
VDR	Forward: 5′- GAATGTGCCTCGGATCTGTGG-3′
	Reverse: 5′-ATGCGGCAATCTCCATTGAAG-3′
Alp	Forward: 5′- CCAACTCTTTTGTGCCAGAGA-3′
	Reverse: 5′-GGCTACATTGGTGTTGAGCTTTT-3′
Col-I	Forward: 5′- TAAGGGTCCCCAATGGTGAGA-3′
	Reverse: 5′-GGGTCCCTCGACTCCTACAT-3′
OCN	Forward: 5′- CTGACCTCACAGATCCCAAGC-3′
	Reverse: 5′-TGGTCTGATAGCTCGTCACAAG-3′
