# Supplementary figures and images for: Nodakenin Ameliorates Ovariectomy-Induced Bone Loss by Regulating Gut Microbiota
Source: Molecules. 2024 Mar 11;29(6):1240. doi: 10.3390/molecules29061240 (PMC10976110; doi:10.3390/molecules29061240)

**A**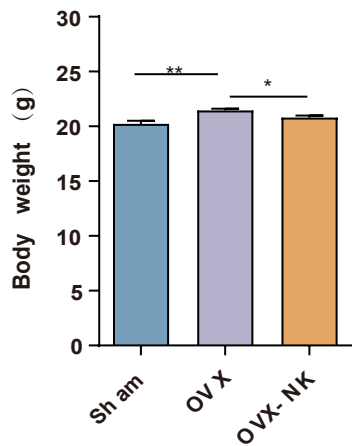**B**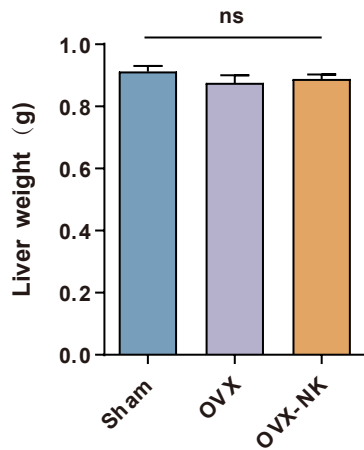**C**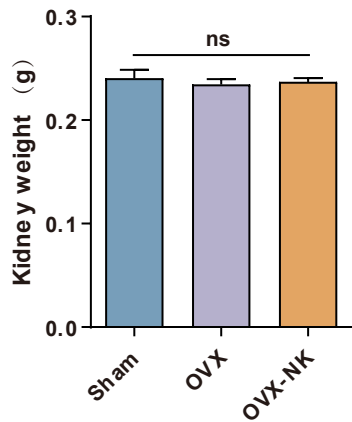**D**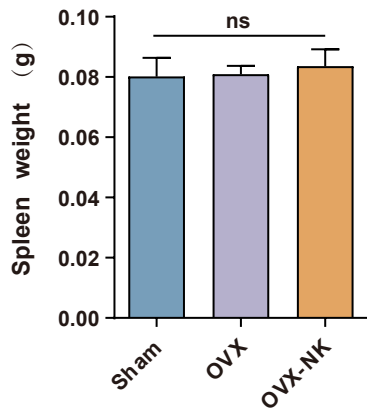

Supplement: Supplementary file 1 [file molecules-29-01240-s001.zip › Supplementary Figure S1.pdf]

**A**

Rarefaction Curves

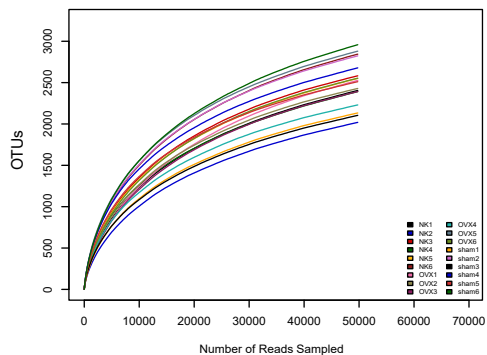**B**

Shannon-Wiener Curves

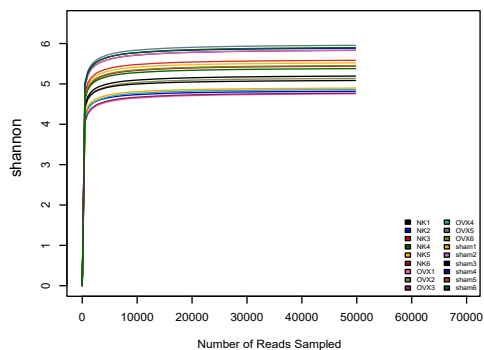

Supplement: Supplementary file 1 [file molecules-29-01240-s001.zip › Supplementary Figure S2.pdf]
